# Valuation effects of U.S. monetary policy tightening: The roles of foreign exposure

**DOI:** 10.1371/journal.pone.0351157

**Published:** 2026-06-11

**Authors:** Xin Fang, Xingyue Peng

**Affiliations:** 1 School of Management, Hefei University, Hefei, China; 2 College of Business, Shanghai University of Finance and Economics, Shanghai, China; University of Tunis El Manar Faculty of Economic Sciences and Management of Tunis: Universite de Tunis El Manar Faculte des Sciences Economiques et de Gestion de Tunis, TUNISIA

## Abstract

Starting in 2022, the United States launched a new round of monetary policy tightening, adopting a dual-track strategy of sustained interest rate hikes and balance sheet reduction. These measures have generated significant spillover effects on China’s stock market. Against this backdrop, this paper employs an event study and regression analysis to investigate the short-term market response of Chinese A-share listed firms to U.S. monetary tightening shocks. The analysis captures firms’ overseas exposure from three dimensions—trade, investment, and financial channels. Empirical results reveal that trade exposure is positively associated with cumulative abnormal returns (CARs), while both investment and financial exposures exert significant negative effects. The paper further discusses the differentiated moderating mechanisms of managerial resource allocation capacity and VC background across the three types of overseas exposures. Heterogeneity analysis shows that the influence of overseas exposure on market performance varies by firms’ technological intensity. Firms with high investment exposure—particularly those involved in foreign ownership, cross-border M&A, and greenfield investment—perform worse under policy shocks.

## 1. Introduction

As the world’s most influential monetary authority, the Federal Reserve’s policy adjustments are widely regarded as a key driver of the global financial cycle. Its tightening measures—particularly interest rate hikes—have far-reaching impacts on international capital flows, global liquidity, and financial market stability. These shocks are transmitted to other economies through multiple channels, including cross-border capital movements, exchange rate fluctuations, financial market linkages, and trade transmission mechanisms [1; Albrizio et al., 2020; 2]. As the world’s second-largest economy, China has experienced sustained export-driven growth in recent years. According to data from China’s General Administration of Customs, the country’s export volume exceeded RMB 25 trillion in 2024, marking a 7.1% year-on-year increase. Meanwhile, China has transitioned from a net capital importer to a net capital exporter, with outward foreign direct investment (OFDI) expanding rapidly. In terms of external financing, U.S. dollar-denominated debt remains the dominant component of Chinese firms’ foreign currency liabilities, accounting for approximately 80% of the total according to the State Administration of Foreign Exchange. Consequently, Chinese firms are deeply embedded in global economic activities through export trade, OFDI, and foreign currency debt financing, making them increasingly vulnerable to spillover risks from U.S. monetary tightening.

Although existing studies have examined the impact of overseas business activities on firm performance from the perspective of corporate internationalization, the connection between internationalization and external monetary shocks remains insufficiently explored. Prior research on international business activities has primarily focused on the positive effects of export trade, outward foreign direct investment (OFDI), and cross-border mergers and acquisitions on firms’ long-term performance, innovation capability, and resource acquisition. However, limited attention has been paid to whether overseas operations may instead become an important source of risk exposure under the background of continuous U.S. interest rate hikes, global liquidity tightening, and deteriorating external financial conditions. In fact, different types of overseas exposure correspond to distinct risk transmission mechanisms. Firms with higher trade exposure generally possess stronger export competitiveness and may obtain certain buffering benefits from exchange rate fluctuations [3]. In contrast, firms with higher investment exposure, due to their involvement in overseas asset allocation, cross-border operations, and international capital deployment, are more vulnerable to rising uncertainty in foreign markets and asset revaluation risks [4]. Meanwhile, firms with higher financial exposure face increasing U.S. dollar debt burdens, exchange rate depreciation pressure, and tightening external financing constraints [5]. Therefore, U.S. monetary policy tightening may not exert homogeneous effects on all internationalized firms, and different forms of overseas exposure may lead to significantly heterogeneous market responses. Nevertheless, the existing literature largely treats corporate internationalization as a single-dimensional concept, lacking a systematic decomposition of overseas exposure into trade, investment, and financial dimensions. Moreover, few studies have examined the short-term valuation effects of U.S. monetary policy adjustments on firms from the perspective of event shocks.

Therefore, our research takes the date of the September 2022 FOMC meeting as the event day, we systematically examine the response mechanisms through which firms’ overseas exposure—across three dimensions of trade exposure, investment exposure, and financial exposure—reacts to shocks from U.S. monetary policy. The study shows that trade exposure has a significantly positive effect on cumulative abnormal returns, whereas firms with higher investment exposure and higher financial exposure face greater adverse impacts under U.S. policy tightening. The theoretical contributions of this study are threefold. First, this study enriches the conventional research paradigm on the international spillover effects of U.S. monetary policy by deepening the theoretical framework of cross-border monetary transmission mechanisms under open-economy conditions. While the extant literature predominantly focuses on the macroeconomic channels—such as capital flows, exchange rate fluctuations, and financial market volatility [2; 6; 1]—this paper shifts the analytical lens to the firm level. By employing the event study methodology, we quantify the short-term market impact of Federal Reserve policy tightening on Chinese A-share listed firms and identify the pivotal role played by overseas exposure in shaping firms’ differential responses. Second, the paper extends the theory of firms’ internationalization strategies from the perspective of the global financial cycle. Existing literature largely emphasizes the long-term performance outcomes of internationalization [7], yet it underexplores how heterogeneous internationalization patterns mediate firms’ market responses to external financial shocks. By constructing a multidimensional measure of external risk exposure, this study proposes a novel analytical framework to capture how overseas exposure affects the transmission of global financial shocks. Third, this paper offers an innovative exploration of how VC involvement moderates the relationship between overseas exposure and market performance during episodes of U.S. monetary policy tightening, which pushes the boundary of the literature on venture capital by embedding it within the broader discourse of firm internationalization and macroeconomic volatility, offering novel insights into the role of VC in the global business cycle.

## 2. Theoretical analysis and research hypotheses

### 2.1. Literature review

Our study systematically reviews the relationship between foreign exposure and firm performance. First, at the level of trade exposure, prior studies primarily focus on the impact of export activities on firm performance and survival. Existing evidence suggests that moderate diversification of export destinations helps improve firm profitability [3], while the relationship between international experience and export performance exhibits a nonlinear pattern [8]. At the same time, export activities have a “double-edged sword” effect on firm survival: they may increase operational risks in the short term, but enhance long-term survival through productivity improvement [9].

Second, at the level of investment exposure, research mainly examines the performance and innovation effects of outward foreign direct investment (OFDI) and cross-border mergers and acquisitions (M&As). In general, OFDI can enhance firm performance and innovation capability through international learning, technology acquisition, and knowledge spillovers, although these effects are contingent upon industry globalization pressure, firm resource capabilities, and ownership characteristics [4; 10; 11]. Furthermore, OFDI directed toward developed countries is more conducive to improving firms’ innovation performance [12; 13]. Meanwhile, cross-border M&As promote innovation capability through knowledge integration and technological synergies. In particular, digital acquisitions, technology-oriented acquisitions, and strategic and organizational fit can significantly enhance firms’ innovation performance [14; 15; 16].

Finally, at the level of financial exposure, studies indicate that foreign currency debt significantly increases firm value volatility and financial crisis risk, and may negatively affect long-term firm output [17; 5]. Overall, foreign exposure may improve firm performance through resource acquisition, knowledge spillovers, and international learning, while simultaneously increasing operational and financial risks due to greater exposure to external uncertainties.

### 2.2. Trade exposure and corporate performance under U.S. monetary policy tightening

Trade exposure denotes the extent to which a company is embedded within the global trading framework, commonly quantified by the ratio of exports to total sales. Adjustments in U.S. monetary policy can significantly impact firms with high trade exposure through various international trade channels, encompassing exchange rate fluctuations, shifts in financing costs, and contractions in global demand. Previous research has indicated that countries with higher export multipliers are generally more susceptible to the spillover effects of shocks in U.S. monetary policy.

On the one hand, in the short run, U.S. interest rate increases frequently trigger exchange rate movements that bolster the international price competitiveness of Chinese exports. This phenomenon cultivates more optimistic earnings expectations for export-oriented companies. To capitalize on excess returns stemming from exchange rate volatility, investors are more prone to boost their stakes in firms with significant trade exposure. For companies operating in sectors producing necessity goods or high-value-added products with relatively inelastic demand, export orders are likely to stay stable in the short term despite external uncertainties and global market turbulence, thereby partially offsetting the adverse effects of monetary shocks. On the other hand, the “learning-by-exporting” effect substantially enhances a firm’s risk resilience, especially when firms demonstrate high export dependence, large scale, low financing constraints, diversified ownership structures, and varied trade modalities [18; 19]. Empirical evidence also suggests that export-oriented business strategies can effectively prolong a firm’s lifespan. Specifically, export activities have been found to extend a firm’s average life expectancy by roughly 0.63 years. Moreover, multi-product exporters benefit from higher survival probabilities [20], and the synergy between exporting and innovation positively contributes to total factor productivity. In this regard, greater trade exposure can fortify a firm’s resilience by expanding market diversification and enhancing innovation capacity. Based on the above theoretical considerations, this study proposes the following hypothesis:

H1. Firms with higher trade exposure experience less negative impact on market performance during U.S. monetary policy tightening shocks.

### 2.3. Investment exposure and corporate performance under U.S. monetary policy tightening

Within the context of an open economy, this study characterizes investment exposure as the degree to which a firm is integrated into the global capital allocation system and international production networks, typically evidenced through outward foreign direct investment (FDI) activities, including cross-border mergers and acquisitions (M&As) and greenfield investments. In recent years, China has progressively transitioned from being a net capital importer to a net capital exporter. This structural evolution underscores the escalating strategic significance of outbound investment in the business operations of Chinese firms, while simultaneously heightening their susceptibility to volatility in global financial markets.

The tightening of U.S. monetary policy, particularly through interest rate hikes by the Federal Reserve, tightens global liquidity conditions and elevates financing costs for firms in emerging markets [6]. Furthermore, the capital retraction effect linked to U.S. policy shocks exacerbates capital outflow pressures in these markets. These spillover effects are transmitted not only through macroeconomic channels but also via the foreign subsidiary networks of multinational enterprises [21]. From the perspective of the interest rate channel, the tightening of U.S. monetary policy directly increases the financing costs of overseas investment projects and raises barriers to capital access [22; 23]. This is particularly detrimental to highly leveraged investment projects, as their expected returns are significantly diminished by higher financing costs, thereby undermining the profitability and sustainability of firms’ overseas assets [7]. Empirical evidence, employing propensity score matching and difference-in-differences methodologies, demonstrates that cross-border M&As augment firms’ overall risk exposure, especially when host countries exhibit high political risk [24]. Elevated environmental uncertainty in host countries significantly hampers the performance of outward FDI, particularly for greenfield investments. Given that greenfield projects necessitate long-term capital commitments and involve extended payback periods, changes in financing conditions—according to transaction cost theory—can impose severe liquidity constraints on firms, jeopardizing their ability to maintain stable operations in host markets [25]. Additionally, the confidence channel serves as a direct mechanism through which investment exposure influences firm performance amid monetary shocks. When the Federal Reserve adopts a contractionary policy, global investors—driven by risk aversion—tend to reallocate capital towards U.S. Treasury bonds and other dollar-denominated assets with higher liquidity and lower risk. This flight-to-safety behavior can exacerbate capital withdrawal risks for Chinese firms engaged in overseas investments. Based on the above analysis, this study proposes the following hypothesis:

H2. Firms with higher investment exposure experience more severe negative impacts on market performance during U.S. monetary policy tightening shocks.

### 2.4. Financial exposure and corporate performance under U.S. monetary policy tightening

In the framework of an open economy, financial exposure generally denotes the magnitude of a firm’s liabilities denominated in foreign currencies. Following recent policy reforms by the National Development and Reform Commission and the State Administration of Foreign Exchange, which have relaxed restrictions on cross-border financing by domestic entities, foreign currency debt has assumed an increasingly prominent role in firms’ capital structures. While certain studies have indicated that borrowing in foreign currencies can reduce firms’ financial costs and alleviate financing constraints, thereby fostering innovation and production activities [26], this financing approach can also expose firms to significant currency mismatch risks, particularly amid heightened global economic uncertainty.

Under a tightening monetary policy regime orchestrated by the U.S. Federal Reserve, characterized by a contraction in global liquidity, financial exposure exerts substantial downward pressure on firm performance. On the one hand, despite the potential for foreign currency debt to offer lower nominal interest rates, exchange rate depreciation—especially a weakening of the RMB—can trigger substantial translation losses, thereby negating the cost advantage of lower foreign interest rates. On the other hand, for firms with substantial foreign currency liabilities, rate hikes by the Fed elevate the cost of dollar-denominated borrowing, effectively increasing their debt-servicing burden [27]. Foreign currency loans influence firm performance by modifying debt structures and financial leverage [28]. The tighter a firm’s financing constraints, the more pronounced the leverage spillover effects from U.S. monetary policy become. If firms fail to adequately hedge against exchange rate volatility, adjust their debt structures, or secure lower-cost financing channels, they may confront severe liquidity shortages, potentially disrupting their operations and deteriorating market performance [2]. From the vantage point of credit risk, firms with high financial exposure that experience intensified debt-servicing stress may face credit rating downgrades. Such downgrades can precipitate a credit contraction effect, further raising firms’ borrowing costs and undermining their long-term capital allocation efficiency and market competitiveness [D’Mello and Toscano, 2020]. Based on the above analysis, this study proposes the following hypothesis:

H3. Firms with higher financial exposure experience more severe negative market performance under U.S. monetary policy tightening shocks.

## 3. Research design

### 3.1. Data sources

This study constructs its dataset from multiple authoritative sources. Export data are obtained from the “Comprehensive Import and Export Indicators of Listed Firms” within the CCD Customs Import-Export Database. Data on cross-border mergers and acquisitions are sourced from the Thomson SDC Platinum Global M&A Database, while greenfield investment information is extracted from the fDi Markets database. These investment data are then matched by firm name to the CSMAR database of Chinese A-share listed companies. Foreign currency debt data are compiled by combining information from the Wind and iFinD financial terminals, along with the notes to financial statements in listed firms’ annual reports. Data on venture capital involvement are manually collected and cross-verified using listed firms’ annual reports, the CVSource database, and the PEdata platform to ensure maximum coverage and reliability. The remaining firm-level financial and governance data are primarily drawn from the CSMAR database. To ensure the validity and reliability of the empirical analysis, the raw dataset is processed through the following steps: First, firms in the financial and real estate sectors are excluded due to their unique regulatory and financial characteristics, which may bias the empirical results. Second, firms designated as ST or *ST are excluded, as they are typically subject to substantial operational or financial distress. Third, observations with severe missing data are removed to ensure data completeness and analytical robustness. Fourth, to mitigate the influence of extreme values on the estimation results, all key continuous variables are winsorized at the 1st and 99th percentiles.

### 3.2. Variable definitions

#### 3.2.1. Independent variables.

Firstly, Trade Exposure (TE). This paper focuses on fluctuations in market demand under external shocks. Meanwhile, since the pathways through which imports affect firms are complex and difficult to identify, incorporating imports into trade exposure may introduce additional noise. Therefore, this paper uses export exposure as a proxy variable for trade exposure. In the benchmark regression, this paper adopts a firm’s export dependence ratio as the measurement indicator for trade exposure [Ma et al., 2025], which is defined as the ratio of a firm’s total export value to its main business revenue in 2021. In the robustness tests, the natural logarithm of a firm’s total export value in 2021 is used as an alternative indicator. Data on a firm’s main business revenue are sourced from the CSMAR database, while data on a firm’s exports are obtained from the “Comprehensive Indicators Database of Customs Import and Export for Listed Companies” within the CCD Customs Import and Export Database, which integrates authentic customs import and export trade data between China and over 200 countries and regions worldwide. However, since the latest customs import and export data for listed companies only extend to 2017, to mitigate potential biases arising from directly using the 2017 export data, this paper employs industry export growth rates to estimate firms’ export situations in 2021. The specific calculation formula is as follows. Here, $g_{industry(i)}$ represents the export growth rate of the industry to which firm i belongs, with data sourced from the sector-specific export commodity value indices on the iFinD financial terminal. By allocating the industry export growth rates from 2018 to 2021 to individual firms, this approach not only reflects industry-level export changes induced by dynamic external environments such as trade frictions, global economic fluctuations, and the COVID-19 pandemic during this period but also preserves the relative scale differences among sample firms.


$$\begin{array}{c} \begin{array}{c} {export}_{i,\text{2}\text{0}\text{2}\text{1}}={export}_{i,\text{2}\text{0}\text{1}\text{7}}\times \prod_{t=\text{2}\text{0}\text{1}\text{8}}^{\text{2}\text{0}\text{2}\text{1}}\left(\text{1}+g_{industry(i),t}\right) \end{array} \end{array}$$ (1)

Second, Investment Exposure (IE). Investment exposure is proxied by a firm’s outward foreign direct investment (OFDI), calculated as the natural logarithm of the sum of its cross-border mergers and acquisitions (M&A) and greenfield investment amounts. In the baseline regression, cross-border M&A data are obtained from the Thomson SDC Global M&A Database, which provides detailed information such as transaction values, deal timing, and acquirer/target identities. Greenfield investment data are sourced from the fDi Markets database under the Financial Times, which covers global greenfield investment projects since 2003, including information on investment size, destination countries, and job creation. Following the methodology of Chen and Nie [29], this study compiles data on cross-border M&A and greenfield investments by Chinese listed firms from 2003 to 2021. Transactions involving offshore entities registered in tax havens such as the Cayman Islands, British Virgin Islands, and Bermuda are excluded. The remaining records are matched with firm names in the CSMAR database and manually verified. The final dataset aggregates each firm’s cross-border M&A and greenfield investment values over the sample period. For robustness tests, the number of overseas subsidiaries is used as an alternative measure of investment exposure. These data are obtained from the “Composition of Corporate Group Subsidiaries” section of annual reports, where the names and registration locations of each subsidiary are used to identify overseas entities.

Third, Financial Exposure (FE). This study adopts the scale of foreign currency debt as a proxy for financial exposure. Following Kim [30], the main specification measures financial exposure as the ratio of foreign currency-denominated debt to total assets at the end of 2021. For robustness checks, the ratio of foreign currency debt to total liabilities is used to capture the degree of foreign currency financing reliance. Data on total assets and total liabilities are sourced from the CSMAR database, while foreign currency debt information is extracted from both Wind and iFinD financial terminals. Given that some firms do not disclose currency composition in a standardized format, the final dataset is manually cross-validated using the notes to financial statements in annual reports. Foreign currency debt includes monetary items such as short-term borrowings, current portion of long-term liabilities, bonds payable, and long-term borrowings denominated in foreign currencies. All amounts are converted into RMB equivalents.

#### 3.2.2. Dependent variable.

Although alternative indicators such as buy-and-hold abnormal returns (BHAR), Tobin’s Q, and return on assets (ROA) are widely employed in the literature to evaluate firm performance, these measures are less suitable for the present study. Specifically, Tobin’s Q and ROA primarily capture medium- to long-term operational or valuation outcomes and are strongly influenced by firms’ accounting policies, investment cycles, and managerial decisions. As such, they are unable to isolate the immediate valuation effects triggered by exogenous monetary policy shocks. Similarly, BHAR is more appropriate for examining long-horizon stock performance, but it may incorporate substantial noise from subsequent macroeconomic events and firm-specific developments, thereby weakening identification of the short-term spillover effects of U.S. monetary policy tightening. By contrast, cumulative abnormal returns (CARs) derived from the event study methodology are particularly appropriate for capturing investors’ immediate reassessment of firm value surrounding a specific policy announcement. on one hand, by reasonably selecting the estimation period and the event window, the event study methodology effectively mitigates the confounding effects of other non-target factors on market movements, thereby enabling a more precise measurement of the spillover effects of monetary policy events. On the other hand, from the perspective of capturing spillover effects in real time, the event study methodology can reflect the rapid responses of key variables to policy events.

Therefore, our study adopts the event study methodology, using announcements by the Federal Open Market Committee (FOMC) of the U.S. Federal Reserve as a proxy for U.S. monetary policy shocks, to examine their spillover effects on Chinese corporate performance. The monetary policy surprise indicator refers to the unanticipated change in expectations of the federal funds rate, measured through financial market instruments shortly after the Federal Open Market Committee (FOMC) policy announcements. A positive value (i.e., a hawkish surprise) indicates that the actual interest rate hike exceeds market expectations [31]. The last column of the table ranks the observation periods in descending order based on the monetary policy surprise indicator values, while excluding periods with zero interest rate hikes or negative monetary policy surprises (i.e., dovish surprises).

The monetary policy surprise indicator, namely MPS (Monetary Policy Surprise), refers to the unexpected change in the federal funds rate measured through financial market instruments within the high-frequency event window surrounding FOMC policy announcements [32; 31]. A positive value, referred to as a hawkish surprise, indicates that the actual rate hike is stronger than market expectations, whereas a negative value, referred to as a dovish surprise, implies that the degree of monetary easing exceeds market expectations. A larger absolute value indicates a greater degree of policy surprise, suggesting a significant deviation between the FOMC announcement and prior market expectations, which in turn triggers rapid adjustments in financial asset prices. By contrast, when the monetary policy surprise indicator approaches zero, it suggests that the Federal Reserve has communicated effectively and that the market has already formed clear expectations regarding FOMC policy decisions, resulting in limited incremental information contained in the announcement. According to [32], the monetary policy surprise indicator is measured as follows:


$$\begin{array}{c} \begin{array}{c} {MPS}_{t}=i_{t}-\;E\left[i_{t}|x_{t},H_{t-\text{1}}\right]=\left(\alpha_{t}-a_{t}\right)x_{t}+\varepsilon_{t} \end{array} \end{array}$$ (2)

$i_{t}$ denotes the policy interest rate (or interest rate path) actually decided by the FOMC, and $E\left[i_{t}|x_{t},H_{t-\text{1}}\right]$ represents the market’s forecast of the interest rate based on available information. $\alpha_{t}$ and $a_{t}$ denote the Federal Reserve’s actual responsiveness to economic conditions and the market’s expected responsiveness of the Federal Reserve, respectively. $x_{t}$ represents macroeconomic state variables such as the output gap, while $\varepsilon_{t}$ captures structural monetary policy shocks.

**Fig 1:**
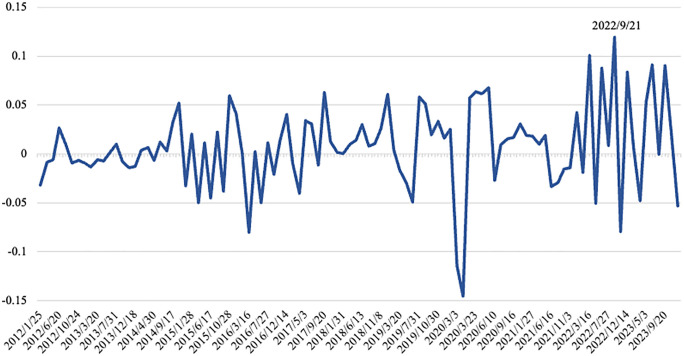
High-frequency monetary policy surprises.

The Fig 1 illustrates the recent trend of high-frequency monetary policy surprise values reported on the official website of the Federal Reserve Bank of San Francisco. The maximum value corresponds to the policy announcement date of the September 22, 2022 FOMC meeting (September 21, 2022, U.S. local time).

Among these, the September 2022 FOMC meeting announcement conveyed the strongest signal of monetary policy tightening, as the actual interest rate hike most significantly exceeded market expectations.

Therefore, this study selects the cumulative abnormal returns (CARs) of firms calculated around the event date of the Federal Reserve’s policy statement release on September 22, 2022 (local time: September 21, 2022) as the dependent variable for the benchmark regression. Additionally, the FOMC meeting announcement date in March 2022, which registered the second-highest monetary policy surprise value after September 2022 (see Table 1), is chosen as an alternative event date for robustness testing.

**Table 1:** Overview of U.S. Monetary Policy Tightening (2022–2023).

Phase	FOMC Meeting Date	Rate Hike (bps)	Federal Funds Rate Ceiling (%)	Balance Sheet Operations (Billion USD)	Unexpected Monetary Policy Indicator Values	Rank
1	2022/03/16	25	0.5	0	0.1021	2
2	2022/05/04	50	1	−470	−0.0460	–
3	2022/06/15	75	1.75	−950	0.0884	3
4	2022/07/27	75	2.5	−950	0.0024	8
5	2022/09/21	75	3.25	−950	0.1084	1
6	2022/11/02	75	4	−950	−0.0807	–
7	2022/12/14	50	4.5	−950	0.0836	4
8	2023/02/01	25	4.75	−950	0.0026	7
9	2023/03/22	25	5	−950	−0.0537	–
10	2023/05/03	25	5.25	−950	0.0544	5
11	2023/06/14	0	5.25	−950	0.0967	–
12	2023/07/26	25	5.5	−950	0.0053	6
13	2023/09/20	0	5.5	−950	0.0924	–

Note: The data is sourced from [31] and the official website of the Federal Reserve.

Following the general framework of the event study methodology: Firstly, the analysis centers on the spillover effects of the Federal Reserve’s monetary policy announcement on September 22, 2022 (September 21, U.S. local time), which is defined as the event date (t = 0). To avoid overlapping influences from multiple events due to excessively long event windows, the study sets the maximum event window as 10 days before and after the event date (−10, +  10). An estimation window of 190 days prior to the event window is established to isolate the impact of historical information. Secondly, Following Chodorow-Reich [33], we first calculate firms’ normal returns using the CAPM, we next derive the Abnormal Return (AR) during the event window as the difference between actual returns and normal returns, and we finally aggregate these ARs to measure the total impact over the event window, yielding the Cumulative Abnormal Return (CAR).


$$\begin{array}{c} \begin{array}{c} {\text{CAR}}_{\text{i}}\left({\text{t}}_{\text{1}},{\text{t}}_{\text{2}}\right)=\sum_{\text{t}={\text{t}}_{\text{1}}}^{{\text{t}}_{\text{2}}}{\text{AR}}_{\text{it}}=\sum_{\text{t}={\text{t}}_{\text{1}}}^{{\text{t}}_{\text{2}}}\left[\left({\text{R}}_{\text{it}}-{\text{R}}_{\text{ft}}\right)-\beta_{\text{i}}\left({\text{R}}_{\text{mt}}-{\text{R}}_{\text{ft}}\right)\right]\; \end{array} \end{array}$$ (3)

Where (${\text{T}}_{\text{1}}$,${\text{T}}_{\text{2}}$) denote the event window under examination, ${\text{T}}_{\text{1}}<{\text{t}}_{\text{1}}\leq {\text{t}}_{\text{2}}\leq {\text{T}}_{\text{2}}$. The term ${\text{CAR}}_{\text{i}}\left({\text{t}}_{\text{1}},{\text{t}}_{\text{2}}\right)$ represents the cumulative abnormal return (CAR) for firm i over the interval (${\text{T}}_{\text{1}}$,${\text{T}}_{\text{2}}$). ${\text{R}}_{\text{it}}$ is the individual stock return of firm i during (${\text{T}}_{\text{1}}$,${\text{T}}_{\text{2}}$), ${\text{R}}_{\text{mt}}$ is the aggregate A-share market return, ${\text{R}}_{\text{ft}}$ is the risk-free rate, $\beta_{\text{i}}$ is the coefficient estimated using firm i’s stock returns and the market return during the estimation window.

### 3.3. Model construct

To examine the impact of overseas exposure on corporate performance under the tightening of U.S. monetary policy, this paper constructs the following empirical models:


$$\begin{array}{c} \begin{array}{c} {CAR}_{i}(-\text{1},\text{1})=\alpha_{\text{0}}+\alpha_{\text{1}}{TE}_{i}+\mu {Control}_{i}+\varphi_{j}+\varphi_{p}+\varepsilon_{i}\; \end{array} \end{array}$$ (4)

$$\begin{array}{c} \begin{array}{c} {CAR}_{i}(-\text{1},\text{1})=\alpha_{\text{0}}+\alpha_{\text{1}}{IE}_{i}+\mu {Control}_{i}+\varphi_{j}+\varphi_{p}+\varepsilon_{i}\; \end{array} \end{array}$$ (5)

$$\begin{array}{c} \begin{array}{c} {CAR}_{i}(-\text{1},\text{1})=\alpha_{\text{0}}+\alpha_{\text{1}}{FE}_{i}+\mu {Control}_{i}+\varphi_{j}+\varphi_{p}+\varepsilon_{i}\; \end{array} \end{array}$$ (6)

where ${CAR}_{i}(-\text{1},\text{1})$ denotes the cumulative abnormal return of A-share listed firms in China during the event window surrounding the U.S. Federal Reserve’s monetary policy announcement on September 22, 2022 (September 21, U.S. local time). Overseas exposure is captured by three proxies: trade exposure (TE), investment exposure (IE), and financial exposure (FE). $\;{Control}_{i}$ represents a set of firm-level control variables: (1) enterprise age (EA) (2) enterprise size (ES) (3) Total liabilities at the end of the period / Total assets at the end of the period (ALR) (4) Net cash flow from operating activities / Total assets at year-end (CF) (5) Financial expenses / Operating revenue (FC) (6) (Sum of short-term and long-term borrowings) / Operating revenue (EFD) (7) Takes the value of 1 if the firm is state-owned, and 0 otherwise (EPR) (8) (Number of independent directors / Total number of board members) × 100% (IDP). $\varphi_{j}$ and $\varphi_{p}$ denote industry and province fixed effects, respectively, and $\varepsilon_{i}$ is the idiosyncratic error term. All continuous variables are standardized, and standard errors are clustered at the industry level.

## 4. Empirical analysis

### 4.1. Baseline regression analysis

The regression results are reported in Table 2. First, trade exposure exhibits a significantly positive effect on CAR (coef = 0.727, P < 0.01), this finding supports H1. The tightening of U.S. monetary policy often triggers a reallocation of global capital flows, leading to an appreciation of the U.S. dollar and a relative depreciation of the Chinese yuan. At this juncture, the price competitiveness of products from China’s export-oriented enterprises is enhanced due to exchange rate fluctuations, which in turn drives investors to improve their expectations regarding the future profitability of such enterprises. Consequently, in the process of asset allocation, investors are more inclined to increase their holdings of stocks in highly export-dependent enterprises that possess global market share and exchange rate advantages, with the aim of obtaining excess returns.

**Table 2:** Benchmark regression results.

Model	(1)	(2)	(3)
Variable	CAR(−1,1)	CAR(−1,1)	CAR(−1,1)
TE	0.727***		
	(0.241)		
IE		−0.066*	
		(0.038)	
FE			−0.523***
			(0.180)
EA	0.422	0.320	0.381
	(0.340)	(0.260)	(0.544)
ES	0.051	0.125*	0.118***
	(0.079)	(0.066)	(0.023)
ALR	0.502	0.525	3.695
	(0.517)	(0.697)	(4.837)
CF	−0.325	1.964*	2.698
	(1.053)	(1.180)	(1.699)
FC	−5.798***	−3.038**	−2.584
	(1.741)	(1.338)	(2.809)
EFD	−0.119	−0.091	−0.215
	(0.178)	(0.137)	(0.287)
IDP	−0.835	−2.175*	−1.390
	(1.465)	(1.126)	(2.362)
EPR	−0.504**	−0.361**	−1.400***
	(0.216)	(0.167)	(0.350)
Industry	Yes	Yes	Yes
Province	Yes	Yes	Yes
Constant	−3.064**	−3.927**	−6.509***
	(1.530)	(1.593)	(2.249)
N	3876	3876	3876
Adjusted R^2^	0.104	0.112	0.085

Notes: ***P < 0.01, **P < 0.05, *P < 0.1; the same applies below.

Second, investment exposure is found to have a significantly negative effect on CAR (coef = −0.066, P < 0.10), this result validates H2. The Fed’s interest rate hikes will worsen the market financing environment and elevate policy uncertainty in host countries, thereby increasing the default risk of corporate investment projects and reducing returns on overseas investments. At this time, corporate strategic adjustments also face higher transaction costs, which will lead investors to adopt a more pessimistic view of the operational risks of enterprises with significant exposure to overseas investments.

Third, financial exposure is negatively associated with CAR at a statistically significant level (coef = −0.523, P < 0.01), it supports H3. The Fed’s interest rate hikes directly result in increased debt interest denominated in U.S. dollars for enterprises with financial exposures, eroding their profit margins. Due to the leverage effect, investors become more concerned about the financial stability of these enterprises. Additionally, as the enterprises face heightened debt repayment pressures, their credit ratings are downgraded, leading to higher financing costs and weakened future growth potential, which in turn affects investor expectations.

### 4.2. Robustness tests

#### 4.2.1. Replacement of dependent variable.

On the one hand, to verify the robustness of the baseline regression results, this study extends the event window and redefines the dependent variable as the cumulative abnormal return (CAR) over a longer window, CAR(−6,6), representing Chinese A-share listed firms’ stock performance during the six trading days before and after the U.S. Federal Reserve’s monetary policy announcement. The empirical results are presented in the Table 3, which is consistent with the research hypotheses.

**Table 3:** Replacement of dependent variable.

	FOMC Meeting Date = 2022/09/21			FOMC Meeting Date = 2022/03/16		
Model	(1)	(2)	(3)	(4)	(5)	(6)
Variable	CAR(−6,6)	CAR(−6,6)	CAR(−6,6)	CAR(−6,6)	CAR(−6,6)	CAR(−6,6)
TE	0.397**			0.708***		
	(0.185)			(0.233)		
IE		−0.060*			−0.046**	
		(0.032)			(0.021)	
FE			−0.162***			−0.682***
			(0.055)			(0.199)
Controls	Yes	Yes	Yes	Yes	Yes	Yes
Industry	Yes	Yes	Yes	Yes	Yes	Yes
Province	Yes	Yes	Yes	Yes	Yes	Yes
Constant	−5.757***	−7.549***	−3.584**	−7.652***	−7.991***	−9.378***
	(1.801)	(1.594)	(1.789)	(2.774)	(2.897)	(3.087)
N	3876	3876	3876	3876	3876	3876
Adjusted R^2^	0.111	0.116	0.110	0.038	0.042	0.032

On the other hand, our study selects the announcement date of the policy-setting meeting on March 17, 2022 (local time in the U.S.: March 16, 2022) as an alternative event date and recalculates the cumulative abnormal returns (CARs) for the day before and after the event to conduct robustness tests. The aim is to exclude potential estimation biases arising from reliance on a specific event date and ensure the reliability of the benchmark regression results. The regression results are presented in the Table 3. Therefore, the key findings of the benchmark regression are not contingent on the selection of a specific event date, further supporting the robustness of the main conclusions.

#### 4.2.2. Replacement of explanatory variables.

To further assess the robustness of the baseline regression results, this study adopts alternative measures for the core explanatory variables. Specifically, the natural logarithm of a firm’s total export value in 2021 is employed as an alternative proxy for trade exposure; the number of overseas subsidiaries listed by a firm is used to capture investment exposure; and the ratio of year-end foreign currency debt to total liabilities is utilized as a proxy for financial exposure. As reported in the Table 4, the results reinforce the validity of the baseline findings.

**Table 4:** Replacement of explanatory variables.

Model	(1)	(2)	(3)
Variable	CAR(−1,1)	CAR(−1,1)	CAR(−1,1)
TE	0.033***		
	(0.011)		
IE		−0.018**	
		(0.008)	
FE			−0.079**
			(0.036)
Controls	Yes	Yes	Yes
Industry	Yes	Yes	Yes
Province	Yes	Yes	Yes
Constant	−7.276***	−6.392***	−9.316**
	(1.537)	(1.559)	(3.746)
N	3876	3876	3876
Adjusted R^2^	0.102	0.117	0.085

### 4.3. Endogeneity test

#### 4.3.1. EBM.

To address potential endogeneity concerns arising from omitted variable bias and sample selection issues, since Propensity Score Matching (PSM) is highly dependent on the specification of the first-stage Logit model, and even minor differences in model specification can significantly impact the matching results, this paper introduces the Entropy Balancing Matching (EBM) method for analysis and validation to effectively circumvent the drawbacks of PSM. Firstly, the sample is divided into a treatment group and a control group based on the mean values of trade exposure, investment exposure, and financial exposure. Then, entropy balancing matching is conducted using all control variables in this paper as covariates to achieve balance in the distribution of covariates between the two groups. Finally, the model is regressed according to the weights derived from entropy balancing matching. After processing with the entropy balancing matching method, the mean values, variances, and skewness of the covariates in the treatment and control groups are quite similar, with notable reductions in differences, indicating an overall satisfactory matching effect. The regression results of the sample after entropy balancing matching are presented in Table 5. The results remain consistent with the baseline regression, which confirms the robustness of our findings.

**Table 5:** EBM test results.

Model	(1)	(2)	(3)
Variable	CAR(−1,1)	CAR(−1,1)	CAR(−1,1)
TE	0.296**		
	(0.132)		
IE		−0.063*	
		(0.035)	
FE			−0.325**
			(0.141)
Controls	Yes	Yes	Yes
Industry	Yes	Yes	Yes
Province	Yes	Yes	Yes
Constant	−5.629***	−9.281***	−8.436***
	(1.323)	(2.435)	(2.476)
N	1614	1305	1256
Adjusted R^2^	0.124	0.157	0.181

#### 4.3.2. 2SLS.

To address potential endogeneity issues, our study employs the average trade exposure (IV1), investment exposure (IV2), and financial exposure (IV3) of other firms in the same industry and year as external instrumental variables. The rationale is as follows: in terms of relevance, firms tend to engage in strategic imitation and benchmarking behavior. If other firms in the same industry significantly increase their trade, investment, or financial exposure in a given year, the focal firm is likely to follow suit. In terms of exclusion restriction, firm performance is primarily the result of firm-specific decisions, and the trade, investment, or financial exposure of other firms does not directly determine a given firm’s performance under U.S. monetary tightening shocks. The first-stage regression results in Table 6 Models 1–3, show that the instrumental variables are significantly positively correlated with the endogenous explanatory variables, with F-statistics exceeding the threshold of 10, confirming the strength and relevance of the instruments. The second-stage regression results in Table 6 Models 4–6, show that the coefficients of the endogenous variables are significantly positive at the 1% level, significantly negative at the 10% level, and significantly negative at the 5% level, respectively—thus providing further support for the hypotheses. Therefore, after addressing endogeneity concerns, the hypothesis testing results in this paper remain robust.

**Table 6:** 2SLS test results.

	First-stage regression			Second-stage regression		
Model	(1)	(2)	(3)	(4)	(5)	(6)
Variable	TE	IE	FE	CAR(−6,6)	CAR(−6,6)	CAR(−6,6)
IV1	0.824***					
	(0.040)					
IV2		0.186**				
		(0.074)				
IV3			0.561***			
			(0.062)			
TE				0.335***		
				(0.047)		
IE					−0.170*	
					(0.090)	
FE						−0.183**
						(0.082)
F	11.231	13.375	18.675			
Controls	Yes	Yes	Yes	Yes	Yes	Yes
Industry	Yes	Yes	Yes	Yes	Yes	Yes
Province	Yes	Yes	Yes	Yes	Yes	Yes
Constant	−1.239***	−2.759***	−1.939***	−8.205***	−8.669***	−8.309***
	(0.120)	(0.132)	(0.129)	(1.116)	(1.158)	(1.113)
N	3876	3876	3876	3876	3876	3876
Adjusted R2	0.119	0.142	0.132	0.151	0.125	0.151

## 5. Further analysis

### 5.1. Extension of investment exposure perspective

In the preceding analysis, firms’ overseas investment exposure was examined primarily from the perspective of outbound capital flows. This section shifts the focus to inbound capital by measuring investment exposure through the shareholding ratio of foreign investors (Fshare), which is sourced from the Wind Financial Terminal. The baseline regression results are presented in Table 7. Firms with higher foreign ownership stakes exhibit significantly worse cumulative abnormal returns during periods of U.S. monetary policy tightening.

**Table 7:** Extension of investment exposure perspective.

	Foreign investor shareholding ratio		Cross-border M&A		Greenfield investment	
Model	(1)	(2)	(3)	(4)	(5)	(6)
Variable	CAR(−1,1)	CAR(−1,1)	CAR(−1,1)	CAR(−1,1)	CAR(−1,1)	CAR(−1,1)
Fshare	−1.819**	−1.157				
	(0.876)	(1.022)				
CBM			−0.073**	−0.014		
			(0.035)	(0.069)		
GI					−0.096**	−0.089*
					(0.039)	(0.047)
VC		0.444***		−0.404		0.331**
		(0.164)		(0.617)		(0.143)
Interaction		−1.600		0.034**		0.281*
		(2.064)		(0.015)		(0.149)
Controls	Yes	Yes	Yes	Yes	Yes	Yes
Industry	Yes	Yes	Yes	Yes	Yes	Yes
Province	Yes	Yes	Yes	Yes	Yes	Yes
Constant	−11.183***	−4.953***	−9.411***	−4.571***	−9.620***	−10.026***
	(3.750)	(1.785)	(3.133)	(1.546)	(3.212)	(3.227)
N	3876	3876	3876	3876	3876	3876
Adjusted R^2^	0.149	0.113	0.099	0.101	0.117	0.102

Furthermore, this study refines the measurement of overseas investment exposure by decomposing it into cross-border mergers and acquisitions (CBM) and greenfield investment (GI). Specifically, CBM is proxied by the natural logarithm of the total value of cross-border M&A transactions from 2003 to 2021, with data obtained from the Thomson SDC Platinum M&A Database. GI is proxied by the natural logarithm of greenfield investment value over the same period, based on data from the fDi Markets database. The regression results in Table 7 show that firms with greater CBM exposure face stronger market headwinds during U.S. monetary tightening episodes. This finding likely reflects increased financial burdens associated with overseas acquisitions amid rising global borrowing costs, coupled with elevated policy uncertainty and exchange rate volatility in host countries, which together dampen investor confidence in the profitability of such firms. As for greenfield investment, its impact on cumulative abnormal returns is also negative and statistically significant. Given the long-term capital commitment characteristic of GI, firms with higher GI exposure are more exposed to prolonged return cycles and intensified liquidity pressures when the external financing environment tightens.

Finally, regarding the moderating mechanism in Table 7, venture capital (VC) involvement does not exert a statistically significant moderating effect on the adverse impact of foreign ownership-based investment exposure. This suggests that VC investors are limited in their ability to mitigate market pressures stemming from large-scale international capital withdrawal. However, VC participation significantly attenuates the negative effects of both CBM and GI during U.S. monetary tightening periods. This indicates that VC functions as “patient capital” by improving corporate governance and enabling strategic capacity, thereby enhancing cross-border firms’ resilience to external monetary shocks.

### 5.2. Test of moderating effect

#### 5.2.1. The moderating role of managerial resource allocation ability.

Drawing on resource dependence theory and upper echelons theory, organizations exhibit a high degree of dependence on environmental resources, and a firm’s ability to effectively acquire and allocate critical resources fundamentally determines its adaptive capacity. Management teams with superior resource allocation ability are able to optimize the structure and efficiency of resource deployment, thereby enhancing the firm’s resilience to external risks. This study further investigates the mechanism through which firms’ overseas exposure affects their market performance under the shock of U.S. monetary policy tightening from the perspective of managerial resource allocation ability. Managerial resource allocation ability concerns how effectively a firm’s resources—such as human, physical, and financial capital—are allocated and utilized to ensure the realization of corporate objectives. This ability requires managers to possess a deep understanding of both internal and external resources, and to allocate them rationally based on actual needs and priorities. Through efficient resource allocation, firms can maximize resource utilization, improve productivity and market competitiveness, and better achieve their developmental goals.

Following Demerjian et al. [34], this paper measures managerial resource allocation ability using a two-stage DEA-Tobit model. Specifically, in the first stage, a Data Envelopment Analysis (DEA) model evaluates the overall efficiency of firms, where the numerator represents Resource Allocation Efficiency (RAE), defined as the ratio of after-tax profit to net assets. The denominator includes net fixed assets (NFA), net R&D expenditures (NRD), goodwill (GW), intangible assets (IA), operating costs (OC), and the sum of selling and administrative expenses (SGA). In the second stage, the residuals obtained from the first-stage model serve as the measure of managerial resource allocation ability (MRAA).


$$\begin{array}{c} \begin{array}{c} MAXFE=\frac{RAE}{\alpha_{\text{1}}NFA+\alpha_{\text{2}}NRD+\alpha_{\text{3}}GW+\alpha_{\text{4}}IA+\alpha_{\text{5}}OC+\alpha_{\text{6}}SGA} \end{array} \end{array}$$ (7)

$$\begin{array}{c} \begin{array}{c} FE=\gamma_{\text{0}}+\gamma_{\text{1}}Size+\gamma_{\text{2}}MS+\gamma_{\text{3}}FSF+\gamma_{\text{4}}Age+\gamma_{\text{5}}CS+\gamma_{\text{6}}FC+\varepsilon \end{array} \end{array}$$ (8)

The regression results are reported in Table 8. The coefficient on the interaction between managerial resource allocation ability and trade exposure is positive and statistically significant. This is because management teams with high allocation ability can better leverage market opportunities arising from trade exposure, optimize supply chains and sales channels, and enhance the firm’s international competitiveness. Similarly, managerial resource allocation ability mitigates the negative effect of investment exposure on firm performance under monetary policy shocks. By accurately assessing the economic environment and firm-specific conditions, high-ability managers can integrate resources more effectively, reducing the sensitivity of capital investment cash flows to financing constraints, minimizing inefficient investment losses, and fostering higher levels of innovation and innovation efficiency. Finally, the moderating effect of managerial resource allocation ability on financial exposure is not significant. Foreign currency debt is typically rigid, and even strong allocation ability cannot alter existing debt costs. Furthermore, liquidity constraints limit managers’ capacity to offset the tightening of external financing conditions through internal reallocations.

**Table 8:** The moderating role of managerial resource allocation ability.

Model	(1)	(2)	(3)
Variable	CAR(−1,1)	CAR(−1,1)	CAR(−1,1)
TE	0.624***		
	(0.060)		
IE		−0.067	
		(0.079)	
FE			−0.307
			(5.322)
MRAA	0.149***	0.177***	0.079*
	(0.022)	(0.012)	(0.046)
Interaction	0.071***	0.023*	0.034
	(0.023)	(0.013)	(0.033)
Controls	Yes	Yes	Yes
Industry	Yes	Yes	Yes
Province	Yes	Yes	Yes
Constant	−6.488***	−7.267***	−9.315***
	(2.086)	(2.554)	(3.211)
N	3876	3876	3876
Adjusted R^2^	0.126	0.108	0.106

#### 5.2.2. The moderating role of venture capital background.

As a vital component of market-oriented capital, venture capital (VC) plays a multifaceted role beyond merely providing funding. It contributes meaningfully to corporate governance, strategic reorientation, and more efficient resource allocation [35]. In the context of U.S. monetary policy tightening, heightened risk premia tend to destabilize capital markets in emerging economies, increase valuation volatility, and induce performance divergence across firms. Venture capital institutions with overseas backgrounds generally possess extensive international networks and global resources. They can assist enterprises in expanding into overseas markets and securing global market collaboration opportunities, thereby alleviating competitive pressure in the domestic market [Guo et al., 2013]; The unique political resources brought by the government background of venture capital institutions help the invested enterprises to learn about policy developments and market information at an earlier stage [Guerini et al., 2016].

Before outlining the measurement of venture capital backgrounds, it is important to clarify the concept of the leading venture capita. The scope of whether a venture capital institution is engaged in independent or joint investment in a company. If the investment is determined to be independent, then the venture capital institution is considered the leading VC institution. In the joint investment, the leading VC institution is identified based on the proportion of equity held by venture capital institution. Specifically, the VC institution with the highest equity proportion is regarded as the leading VC institution. To determine whether a firm receives syndicated venture capital, we first assess VC involvement. We obey the following criteria: Firstly, a listed firm is directly classified as VC-backed if any of its top ten shareholders contain keywords such as “venture capital,” “startup investment,” or “venture capital investment” in their names, ${VCIN\_dummy}_{i}$ is assigned a value of 1. Secondly, if shareholder names include terms like “high-tech investment,” “innovation investment,” “technology investment,” “technical transformation investment,” “information industry investment,” “technology industry investment,” or “high-tech equity investment,” verification step are required: Approach involves consulting authoritative databases, refer to the VC institution lists published annually in the CVsource Investment Database and the Zero2IPO PE Data Tong Database. If a shareholder appears in these lists, the firm is confirmed as VC-backed. Finally, if the top ten shareholders include two or more venture capital firms, they are assigned a value of 1 and considered Syndicated venture capital. Therefore, government-backed venture capital include government-guided funds and central or local state-owned or partially state-owned investment institutions. If the leading VC institution for a company’s shareholders is government-backed, VC_Gov is assigned a value of 1; otherwise 0. Similarly, foreign-backed venture capital institutions include foreign independent investments and Sino-foreign joint ventures. If the leading VC institution for a company’s shareholders is foreign-backed, VC_For is assigned a value of 1; otherwise 0.

The regression results are presented in Table 9. On the one hand, a government-backed VC background amplifies the positive effect of export exposure and, to a certain extent, mitigates the adverse impact of financial exposure, while its effect on investment exposure is not statistically significant. This suggests that VC institutions with government backing, leveraging policy resources and credit endorsement functions, not only help export-oriented firms optimize their market positioning but also provide financing facilitation and liquidity support to firms with high foreign currency-denominated debt. On the other hand, the interaction terms between foreign-backed VC backgrounds and both investment exposure and financial exposure are positive and statistically significant, whereas their moderating effect on trade exposure is insignificant. This indicates that VC institutions with foreign backgrounds, drawing on their cross-border investment experience, governance empowerment, and risk hedging capabilities, not only reduce the adverse impacts faced by firms with investment exposure during monetary tightening cycles but also support firms with high foreign currency-denominated debt through international banking networks and risk management services.

**Table 9:** The moderating role of venture capital background.

Model	(1)	(2)	(3)	(4)	(5)	(6)
Variable	CAR(−1,1)	CAR(−1,1)	CAR(−1,1)	CAR(−1,1)	CAR(−1,1)	CAR(−1,1)
TE	0.676***	0.286**				
	(0.245)	(0.435)				
IE			−0.025*	−0.062**		
			(0.014)	(0.026)		
FE					−0.470**	−0.483***
					(0.215)	(0.173)
VC_Gov	0.211**		0.617		0.416**	
	(0.093)		(0.431)		(0.173)	
VC_For		0.394		0.457**		0.337**
		(0.714)		(0.197)		(0.143)
Interaction	0.012*	0.085	0.081	0.154**	0.086*	0.067**
	(0.007)	(0.587)	(0.146)	(0.067)	(0.048)	(0.030)
Controls	Yes	Yes	Yes	Yes	Yes	Yes
Industry	Yes	Yes	Yes	Yes	Yes	Yes
Province	Yes	Yes	Yes	Yes	Yes	Yes
Constant	−6.123***	−4.524***	−3.673***	−4.249***	−6.755***	−7.919***
	(1.122)	(1.456)	(0.994)	(0.969)	(1.292)	(1.440)
N	3876	3876	3876	3876	3876	3876
Adjusted R2	0.123	0.122	0.144	0.138	0.163	0.137

### 5.3. Heterogeneity analysis

High-tech-intensive firms derive their competitive advantage primarily from technological innovation, characterized by greater complexity and faster technological obsolescence. Following the approach of Brown et al. [36], this study uses the ratio of intangible assets to total assets as a proxy for a firm’s technological intensity. Firms are then categorized into high- and low-tech-intensity groups based on the sample mean. We examine how overseas exposures affect firms’ market performance under U.S. monetary tightening shocks, conditional on technological intensity.

First, the coefficient of trade exposure is significantly positive for both high- and low-tech-intensive firms in Table 10, indicating that firms with greater trade exposure tend to perform better in the stock market during monetary tightening episodes. However, the magnitude of the coefficient is larger for high-tech-intensive firms. This suggests that high-tech firms, benefiting from stronger product pricing power, bargaining strength, and supply chain management capabilities, are more capable of extracting higher value-added returns from global markets. Second, investment exposure exerts a significant negative effect on cumulative abnormal returns in both subgroups, with a larger negative coefficient for low-tech-intensive firms in Table 10. This implies that the adverse effects of U.S. monetary policy shocks on firms’ overseas investment activities are more pronounced among firms with lower technological sophistication. High-tech firms often engage in overseas investments rooted in core technological assets, advanced manufacturing systems, and innovation capabilities, which enjoy high entry barriers and greater market resilience. These attributes help sustain their long-term investment value and investor confidence under external shocks. In contrast, low-tech firms tend to invest in labor-intensive industries with limited value-added potential, making them more vulnerable to financing constraints and declining returns when the global credit environment tightens. Finally, financial exposure has no statistically significant effect on the market performance of high-tech-intensive firms under policy shocks, whereas for low-tech-intensive firms, the coefficient is negative and significant in Table 10. The latter group typically lacks bargaining power in capital markets and faces higher financing costs, leading to mounting financial burdens during periods of monetary contraction. High-tech firms, by contrast, tend to enjoy diversified funding sources—such as venture capital and government subsidies—thanks to their strong innovation capacity and industry competitiveness. Given investors’ relatively stable expectations for their long-term growth, the financial impact of monetary tightening on high-tech firms is not statistically significant.

**Table 10:** Heterogeneity analysis.

	High-tech- intensity	Low-tech-intensity	High-tech- intensity	Low-tech-intensity	High-tech- intensity	Low-tech-intensity
Model	(1)	(2)	(3)	(4)	(5)	(6)
Variable	CAR(−1,1)	CAR(−1,1)	CAR(−1,1)	CAR(−1,1)	CAR(−1,1)	CAR(−1,1)
TE	0.704***	0.666**				
	(0.046)	(0.302)				
IE			−0.185**	−0.263***		
			(0.080)	(0.040)		
FE					0.158	−0.613***
					(6.076)	(0.045)
Controls	Yes	Yes	Yes	Yes	Yes	Yes
Industry	Yes	Yes	Yes	Yes	Yes	Yes
Province	Yes	Yes	Yes	Yes	Yes	Yes
Constant	−5.236**	−4.997**	−6.750**	−6.362***	−3.420**	−4.708**
	(2.621)	(2.383)	(2.795)	(2.238)	(1.599)	(2.057)
N	1227	2649	1227	2649	1227	2649
Adjusted R^2^	0.145	0.106	0.146	0.110	0.178	0.089

## 6. Discussion

### 6.1. Research conclusion

This paper investigates the spillover effects of U.S. monetary policy tightening under open-economy conditions by examining the dynamic relationship between firms’ overseas exposures and their market performance. Using the September 2022 FOMC meeting as the event date, we apply an event-study framework and regression analysis at the micro level to unpack the transmission mechanisms of trade, investment, and financial exposures to U.S. policy shocks. We further extend the investment-exposure perspective by examining foreign investor shareholding, cross-border M&A, and greenfield investment, and explore the role of managerial resource allocation ability and VC background.

Our results show that firms with greater trade exposure enjoy significantly higher cumulative abnormal returns during Fed tightening, whereas those with high investment or financial exposure suffer larger negative shocks. When extending the investment-exposure lens, firms with higher foreign ownership, cross-border M&A, or greenfield investment face greater market pressure during tightening, but venture capital involvement significantly mitigates the negative impacts of M&A and greenfield investment—albeit not of foreign ownership. Management teams with superior resource allocation ability further enhance the trade-exposure advantage and alleviate the investment-exposure loss, but they cannot significantly offset the burden of financial exposure. Government-backed VC background amplifies the positive effect of export exposure and, to a certain extent, mitigates the adverse impact of financial exposure, while its effect on investment exposure is not statistically significant. The interaction term between foreign-backed venture capital and trade exposure is not statistically significant, and to a certain extent, it mitigates the adverse impacts of investment exposure and financial exposure. Finally, heterogeneity analysis reveals that trade exposure benefits are stronger for high-tech-intensity firms, and high-tech firms also experience smaller negative effects from investment exposure and no significant effects from financial exposure.

### 6.2. Practical implications

First, firms should tailor their risk‐mitigation strategies to their specific patterns of international engagement in order to bolster resilience against external financial shocks. While high trade‐exposed firms can secure short‐term competitive gains during Fed tightening, firms with substantial investment or financial exposures face greater market stress. Consequently, exporters should leverage regional trade agreements and the Belt and Road Initiative to diversify their market base and reduce dependence on any single region; firms with heavy investment exposure should moderate short‐term capital outlays abroad and strengthen the self‐sustaining capacity of their overseas operations to mitigate adverse shifts in global financing conditions; and firms with large foreign‐currency liabilities should optimize their currency‐asset‐liability profiles and employ derivatives to hedge exchange‐rate risk.

Second, companies ought to increase their reliance on patient, long‐term capital—such as venture capital, industry funds, and state‐owned investment—to optimize their financing structures and cushion the valuation impact of macroeconomic uncertainty. Long‐horizon investors bring industry expertise and resources that can guide strategic planning and support firms’ international expansion. Third, sustained investment in core technologies is critical for enhancing global bargaining power and long‐term growth prospects. High-tech-intensive firms benefit from innovation-driven barriers to entry that preserve profitability under stress; accordingly, firms should shift from cost-driven to innovation-driven models, deepen patent portfolios, and engage in standards‐setting to strengthen their pricing power and market position.

Finally, strengthening managers’ resource‐allocation ability is essential to maintaining competitive agility in a volatile environment. Management teams with superior allocation skills can precisely integrate and reallocate resources to both mitigate the negative effects of investment exposure and amplify the benefits of trade exposure under external shocks, ensuring efficient decision-making and sustained international competitiveness.

### 6.3. Research limitations and future directions

First, by focusing on immediate stock‐market reactions within a short event window and employing the CAPM to estimate abnormal returns, we do not capture the cumulative, long‐run effects of Fed tightening on firm performance. Future research could apply a Fama–French five‐factor model, in which size, profitability, and investment factors play a larger role in long‐horizon returns, or measure buy‐and‐hold abnormal returns (BHAR) to assess the enduring impact of policy shocks. Second, while we document how different dimensions of overseas exposure influence firms’ sensitivity to monetary tightening, we do not explore the actual hedging instruments and risk‐management strategies that internationally active firms deploy in response to such shocks. Future work could collect firm‐level data on hedging behaviors and evaluate the effectiveness of various tools across different internationalization modes to identify optimal risk‐mitigation paths. Third, our sample is limited to Chinese A‐share listed companies, and cross‐country differences in openness, exchange‐rate regimes, and financial‐market development may yield different transmission mechanisms elsewhere. Extending this framework to other emerging‐market or regional contexts would help clarify how firms’ overseas exposures operate under diverse economic structures and policy environments.
